# Global Transcriptome Profiling of Multiple Porcine Organs Reveals *Toxoplasma gondii*-Induced Transcriptional Landscapes

**DOI:** 10.3389/fimmu.2019.01531

**Published:** 2019-07-03

**Authors:** Jun-Jun He, Jun Ma, Jin-Lei Wang, Fu-Kai Zhang, Jie-Xi Li, Bin-Tao Zhai, Ze-Xiang Wang, Hany M. Elsheikha, Xing-Quan Zhu

**Affiliations:** ^1^State Key Laboratory of Veterinary Etiological Biology, Key Laboratory of Veterinary Parasitology of Gansu Province, Lanzhou Veterinary Research Institute, Chinese Academy of Agricultural Sciences, Lanzhou, China; ^2^Department of Parasitology, College of Veterinary Medicine, Gansu Agricultural University, Lanzhou, China; ^3^Faculty of Medicine and Health Sciences, School of Veterinary Medicine and Science, University of Nottingham, Loughborough, United Kingdom

**Keywords:** *Toxoplasma gondii*, pig, transcriptome, host-parasite interaction, metabolism

## Abstract

We characterized the porcine tissue transcriptional landscapes that follow *Toxoplasma gondii* infection. RNAs were isolated from liver, spleen, cerebral cortex, lung, and mesenteric lymph nodes (MLNs) of *T. gondii*-infected and uninfected (control) pigs at days 6 and 18 postinfection, and were analyzed using next-generation sequencing (RNA-seq). *T. gondii* altered the expression of 178, 476, 199, 201, and 362 transcripts at 6 dpi and 217, 223, 347, 119, and 161 at 18 dpi in the infected brain, liver, lung, MLNs and spleen, respectively. The differentially expressed transcripts (DETs) were grouped into five expression patterns and 10 sub-clusters. Gene Ontology enrichment and pathway analysis revealed that immune-related genes dominated the overall transcriptomic signature and that metabolic processes, such as steroid biosynthesis, and metabolism of lipid and carboxylic acid, were downregulated in infected tissues. Co-expression network analysis identified transcriptional modules associated with host immune response to infection. These findings not only show how *T. gondii* infection alters porcine transcriptome in a tissue-specific manner, but also offer a gateway for testing new hypotheses regarding human response to *T. gondii* infection.

## Introduction

*Toxoplasma gondii* is an obligate intracellular protozoan parasite, which infects nearly one-third of the world human population and all warm-blooded vertebrates (1, 2). There are three infectious stages of this parasite: tachyzoites, bradyzoites-containing tissue cysts, and sporozoites-containing oocysts (the product of sexual reproduction in the intestine of the feline definitive host). Humans acquire infection through ingestion of raw or undercooked meat, such as pork or lamb containing cysts (3, 4). Also, infection can be acquired by ingestion of food contaminated with oocysts or by exposure to soil containing oocysts (5). Despite significant research progress, our understanding of immune-related genes which are substantially involved in the pathogenesis of human toxoplasmosis remains limited. A considerable mass of research has been performed using cell culture models, which improved understanding of the pathogenesis of *T. gondii* infection. However, *in vitro* models cannot fully recapitulate *in vivo* processes.

Animal models can reduce the deficiencies that are inherent to *in vitro* models. Mice (*Mus musculus*) are the most widely model used to study *T. gondii* pathophysiology due to low cost and the availability of specific reagents (6–8). However, mice are less suitable as a model for understanding the transcriptional landscape of other mammalian species that have different transcriptional and genetic backgrounds, such as pig and humans (9). In contrast, transcriptomics and genetic technologies have shown pigs to be genetically and mechanistically relevant to study human conditions (3, 10, 11). Importantly, the common attributes of *T. gondii* infection, such as severity of infection, transplacental transmission, and interferon-gamma-related antiparasitic effector mechanisms are more similar in pigs and humans compared to the same aspects of disease in mice (4, 12–18). These facts suggest that domestic pigs (*Sus scrofa domesticus*) are more relevant model to the study of the pathophysiology of human toxoplasmosis.

Previous studies have provided beneficial but limited insights into the porcine response to *T. gondii* through using methods such as high-throughput sequencing to identify the mRNA and miRNA profiles (19–22), and microarrays (23). Recent research showed that *T. gondii* loads vary across different porcine tissues, with high parasite loads detected in the heart and lungs during acute infection, and in the heart and brain during chronic infection, regardless of the strain of the parasite (24). Therefore, genome-wide comprehensive analysis of the differential responses of pig tissues to *T. gondii* infection is required to elucidate why some porcine tissues vary greatly in their response to *T. gondii* infection.

In this study, the transcriptomic response of five different porcine tissues [brain, lung, liver, spleen, and mesenteric lymph nodes (MLNs)] to experimental *T. gondii* infection was examined using RNA-sequencing (RNA-seq). Our analysis revealed the global transcriptomic changes in relation to *T. gondii* infection at 6 and 18 days after infection. We identified hundreds of differentially expressed transcripts (DETs), infection-specific expression patterns and porcine genes that correlated with *T. gondii* load in infected pig tissues.

## Materials and Methods

### Ethics and Biosafety Statement

The study design was reviewed and approved by the Animal Ethics Committee of Lanzhou Veterinary Research Institute (LVRI), Chinese Academy of Agricultural Sciences (CAAS). The procedures involving animals were carried out in accordance with the Animal Ethics Procedures and Guidelines of the People's Republic of China. Animals were monitored every day for the development of clinical signs of toxoplasmosis. All efforts were made to minimize suffering and to reduce the number of pigs used in the experiment. The potentially infectious clinical and laboratory waste, such as the remaining pig tissues and *T. gondii* oocysts, were decontaminated by autoclaving prior to disposal in accordance with the local institutional health and biosafety policy on the disposal of hazardous waste.

### Animals and Parasite Challenge

Twenty-four, 14-week-old, specific-pathogen-free (SPF), outbred female white pigs were purchased from Beijing Center for SPF Swine Breeding and Management. To confirm the *T. gondii*-free status of pigs before being used in the experiment, pig serum was tested using modified agglutination test (MAT) as described previously (25). The 24 *T. gondii*-seronegative pigs were randomly assigned to eight groups (3 pigs/group), which were housed in separate units. The experimental groups included two control groups (6C_1 and 6C_2) at 6 days post infection (dpi), two control groups at 18 dpi (18C_1 and 18C_2), two infected groups at 6 dpi (6I_1 and 6I_2), and two infected groups at 18 dpi (18I_1 and 18I_2). In the infected groups, each pig was infected orally with 1,000 oocysts of *T. gondii* PYS strain (genotype ToxoDB#9) in 5 ml sterile Phosphate Buffered Saline (PBS, pH 7.4). Pigs in the uninfected (control) group received 5 ml sterile PBS without any oocysts. Pigs from infected and control groups were euthanized at day 6 and day 18 post infection. These two time points post infection were chosen because pig requires 6 days to develop IgM antibodies (indicative of acute infection), whereas IgG antibodies which mark chronic infection develop after 18 dpi (26). Tissue samples were collected from cerebral cortex (thereafter called brain), liver, spleen, lung and MLNs, and were stored separately at −80°C. A total of 200 mg collected from several sites of each organ were used for RNA extraction. Confirmation of *T. gondii* infection in all collected tissues was performed by PCR, as described previously (27).

### Determination of Normalized Parasite Load in Pig Tissues Using Quantitative PCR (qPCR)

DNA of pig tissues was extracted using a TIANamp Genomic DNA Kit (Tiangen Biotech, Beijing, China) according to the manufacturer's recommendations. *T. gondii* B1 gene was used to determine normalized parasite load in pig tissues, and porcine gene coding for 18S rRNA was used to normalize the *T. gondii* B1 DNA to host DNA. Oligonucleotides for amplification of the porcine housekeeping gene 18S rRNA were: 18S-pigF (GCCTGCTGCCTTCCTTG) and 18S-pigR (ATGGTAGTCGCCGTGCC), with an expected product size of ~109 bp. The primers used for detection of *T. gondii* B1 were: B1F (TGCATAGGTTGCAGTCACTG) and B1R (TCTTTAAAGCGTTCGTGGTC) with an expected product size of ~131 bp. The samples with an exponential-amplification curve crossing the threshold were deemed positive for *T. gondii*, whereas samples with no amplification curve for *T. gondii* B1, but amplification of the 18S rRNA gene were considered negative for *T. gondii*. The 2 ^−ΔΔCT^ method [the method can also be displayed as 2^−(CT value of target gene in tested group−CT value of housekeeping gene in^ ^tested group)^/2 ^−(CT value of target gene in control group − CT value of house keeping gene in control group)^] is generally used for calculation of the fold-change of the target genes in infected relative to control samples (28). However, because *T. gondii* gene cannot be detected in the tissue that free of *T. gondii*, we used the cycle threshold (CT) value of target and housekeeping gene to calculate the relative abundance of *T. gondii* B1 gene normalized to pig 18S rRNA gene in each infected tissue using the equation 2^−ΔCT^. − ΔCT = – (CT for the targeted *T. gondii* B1 gene - CT for the pig 18S rRNA gene). qPCR was performed in a Rotor-Gene Q system (QIAGEN, Hilden, Germany) using GoTap® qPCR Master Mix (Promega, Madison, WI, USA). The cycling conditions were 95°C for 5 min followed by 50 cycles of 95°C for 10 s, 60°C for 10 s, 72°C for 15 s; the temperatures of the melt curve analysis ranged from 72 to 95°C to ensure the specificity of the amplification products.

### RNA Extraction and Transcriptome Sequencing (RNA-Seq) Analysis

Total RNA of each collected tissue sample was extracted separately using TRIzol Reagent (Invitrogen China Ltd, Beijing, China) according to the manufacturer's instructions. The residual genomic DNA in the isolated RNA was removed using 20 units of RNase–Free DNase (Ambion, Shanghai, China). The integrity and quantity of RNA samples were tested with Agilent 2100 Bioanalyzer (Agilent Technologies, Santa Clara, CA, USA) and Thermo Scientific™ Nanodrop 2000 (Wilmington, DE, USA), respectively. The RNA-seq analysis was based on two biological replicates per experimental group, and each biological replicate involved pooled RNA samples from three different pigs within each group. Although sample pooling design masks the individual response of pigs, it has been considered as a cost-efficient approach (29–32). Approximately one microgram of total RNA per each pooled sample was used as an input for the construction of mRNA library using IlluminaTruSeq™ RNA Sample Preparation Kit (Illumina, San Diego, CA, USA). The transcriptome libraries were sequenced using IlluminaHiSeq™2000 according to the manufacturer's instructions. Adaptors and low quality sequencing reads were filtered using a quality cutoff score of Q20, which is widely used for quality control analysis (33–37). The resulting clean reads were mapped against the pig (*Sus scrofa domesticus*) reference genome (Sscrofa10.2) using SOAP aligner/SOAP2 software and genomic annotation data file (ref_Sscrofa10.2_top_level.gff3). The level of expression was calculated in units of reads per kilobase per million mapped reads (RPKM) (38). Expression analysis was performed using NOIseq R package (31). Transcripts with |log2 fold-change (FC)| ≥1 and significant value >0.8 were considered differentially expressed, as per the recommendations of the NOIseq. RNA isolation, library construction, RNA sequencing, and computational analysis were performed by BGI-Shenzhen, China.

### Validation of RNA-Seq Data

Thirty-two genes identified by RNA-seq analysis, across all experimental groups, were selected for validation by quantitative reverse transcriptase PCR (qRT-PCR). RNA preparations were those used for RNA-seq experiments at the corresponding time points. cDNA was synthesized from total RNA using the PrimeScript™ II 1st Strand cDNA Synthesis Kit (Takara, Dalian, China) according to the manufacturer's instructions. All qRT-PCR experiments were performed in triplicate, with the housekeeping gene *GAPDH* as a control. The qRT-PCR oligonucleotide primers used in this study are described in Supplementary Table 1. qRT-PCR was performed in Rotor-Gene Q system (QIAGEN, Hilden, Germany) and using GoTap® qPCR Master Mix (Promega, Madison, WI, USA). qRT-PCR cycling conditions were as follows: 95°C for 5 min followed by 50 cycles of 95°C for 10 s, 60°C for 10 s, and 72°C for 15 s; the temperatures of the melt curve analysis ranged from 72 to 95°C to ensure the specificity of qRT-PCR products. The 2^−ΔΔCT^ relative expression method was used to calculate gene expression.

### Gene Ontology (GO) Enrichment and KEGG Analysis

GOseq package (v1.22) in R (www.r-project.org) was used for Gene Ontology (GO) enrichment analysis, such as biological process (BP), cell component (CC), molecular function (MF). Pathway analysis was performed using Kyoto Encyclopedia of Genes and Genomes (KEGG) database. Significantly enriched GO terms or pathways were identified using hypergeometric test followed by FDR correction method (39). The FDR corrected *P* < 0.05 was used as cutoff for the significantly enrichment GO terms or pathways. All differentially expressed transcripts (DETs) were clustered with log2 fold-change of DETs using Gene Cluster 3.0 and Euclidean distance. We used Upset (40) to visualize intersecting sets in order to identify the unique and common DETs across 10 tissue subsets.

### Coexpression Network and Correlation Analysis

We performed coexpression analysis, which has been widely applied to identify genes involved in host-parasite interaction (41–43). The weighted gene correlation network analysis (WGCNA) *R* package (44) was used to establish a correlation matrix between pig mRNA expression and normalized *T. gondii* load in each infected tissue. WGCNA was performed as the network construction and module detection protocol in the WGCNA R package (https://horvath.genetics.ucla.edu/html/CoexpressionNetwork/Rpackages/WGCNA/index.html). RPKMs of all transcripts were used as input data. We have chosen a soft-thresholding power of 14 because this was the lowest power needed to exceed a scale-free topology fit index of 0.75. Days post infection, infection status and normalized *T. gondii* load were used as input traits in the module-trait association analysis. The cluster of highly interconnected genes that share a similar expression pattern was considered as a coexpression module. Multidimensional scaling (MDS) plot was constructed to visualize pairwise relationships specified by a dissimilarity matrix, indicating dissimilarity/similarity based on gene expression data. The hub gene of coexpression module was identified based on a high module membership value or connectivity (i.e., the sum of connection strengths with the other module genes). Generally, the hub genes of specific module are located at the finger tip of MDS plot. For further testing of the predictive performance between the host gene expression and *T. gondii* load, pROC package was used to perform receiver operating characteristic (ROC) curve analysis, and to calculate the area under the ROC curve (AUC), a performance metric, which is generally used for the identification of potential biomarkers. The gene in coexpression module that was significantly correlated with *T. gondii* load, and had significant *P* < 0.05, RPKM > 1, AUC > 0.6, was considered as a host gene significantly correlated with *T. gondii* load (HGSCTG). HGSCTG genomic hotspots were defined on the basis of >5 HGSCTG per 10 Mb genomic region. Gene regulatory networks were reconstructed using the coexpression data and TRRUST database, and were visualized with Cytoscape software. The regulatory transcription factors were identified as the ones that co-express with their target genes. Finally, the relationships between enzymes and substrates were analyzed using the online DrugBank database (https://www.drugbank.ca/).

### Accession Numbers

The RNA-Seq datasets described in this study have been deposited in NCBI Short Read Archive database (https://www.ncbi.nlm.nih.gov/sra) under accession numbers SRR6203124 to SRR6203163.

## Results

### Confirmation of *T. gondii* Infection and Normalized Parasite Load in Pig Tissues

At 6 dpi, all pigs in the infected groups exhibited clinical signs, such as fever and inappetence, whereas pigs in the control groups remained clinically healthy. The brain, liver, spleen, lung, and MLNs of infected pigs were all PCR-positive, whereas no *T. gondii B1* gene amplification was achieved in corresponding tissues from uninfected (control) pigs. Infected lungs showed the highest *T. gondii* load (Supplementary Table 2).

### Transcriptomic Features and Validation of RNA-Seq Results

**Figure 1:**
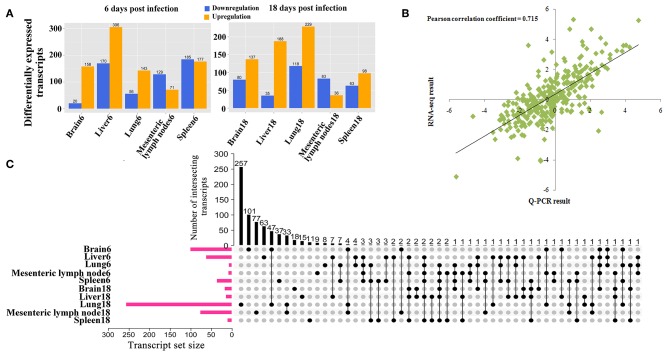
**(A)** The number of differentially expressed transcripts (DETs) across different infected porcine tissues at 6 and 18 days postinfection. **(B)** Validation of RNA-seq results by qRT-PCR. Plot of gene expression (fold change) determined by the RNA-seq (X-axis) and qRT-PCR (Y-axis) of 32 selected genes (Pearson's correlation, *R*^2^ = 0.715, *P* < 0.01). The fold-change of expression was expressed as log2 values. **(C)** UpSet plot showing the sets of differentially expressed transcripts from 10 different tissue samples, including the quantitative analysis of aggregate intersections between tissues. The vertical bars show the number of intersecting transcripts between tissues, denoted by the connected black circles below the histogram. The horizontal bars show the transcript set size.

**Figure 2:**
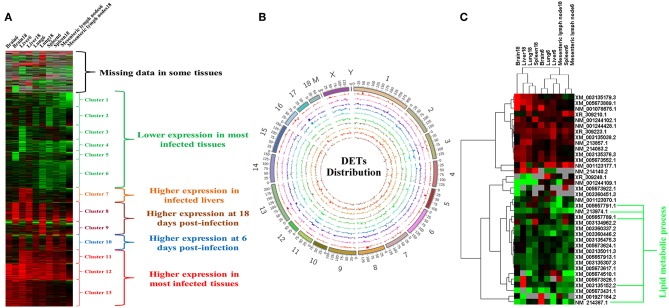
Expression patterns of differentially expressed transcripts (DETs). **(A)** Cluster analysis of all DETs based on the average values of expression of two replicates. **(B)** The distribution of DETs across the chromosomes. From outer to inner circle represent chromosomes, DET bar plots of brain6, brain18, liver6, liver18, lung6, lung18, mesenteric lymph nodes6, mesenteric lymph nodes18, spleen6, and spleen18, respectively. **(C)** Clustering of DETs encoded by X chromosome. Red, green, and gray colors represent upregulated, downregulated and missing (undetected) data, respectively.

The RNA integrity numbers (RINs) of all RNA templates were >8.0. Also, 99% of the reads showed high quality values > Q20, and 90% of the clean reads were up to Q30 (Supplementary Figures 1–3). More than 62 million clean reads were obtained from each tissue sample and more than 32,000 transcripts were detected (Supplementary Table 3). At 6 dpi, 178, 476, 199, 200, and 362 DETs were detected in infected brain, liver, lung, MLNs and spleen, respectively; whereas at 18 dpi, 217, 223, 347, 119, and 161 DETs were found in the infected brain, liver, lung, MLNs and spleen, respectively (Figure 1A). The global Pearson correlation coefficient between qRT-PCR results and RNA-seq results was 0.715 (Figure 1B), suggesting a good agreement between results obtained by the two methods, supporting the validity of the transcriptomic RNA-seq data. After intersecting the differentially expressed transcript sets across tissues, there was not any DET shared across all tissues as shown in the vertical bars and the connected black circles below the histogram (Figure 1C), suggesting a lack of commonly DETs shared across the analyzed tissues. According to Euclidean distance, as shown in Figure 2A, all DETs were clustered into five distinct expression patterns, including (pattern 1) low expression in most infected tissues (downregulated in ≥ 6 tissue samples at 6 and 18 dpi), (pattern 2) high expression in infected liver only, (pattern 3) high expression at 18 dpi, (pattern 4) high expression at 6 dpi, and (pattern 5) high expression in most infected tissues (upregulated in ≥ 6 tissue samples at 6 and 18 dpi). The distribution of DETs across chromosomes is shown in Figure 2B. Three chromosomes encoded most of the DETs: chromosome 2 (110 DETs), chromosome 6 (108 DETs), and chromosome 7 (114 DETs). No DETs were found in the mitochondrial DNA or chromosome Y. However, 37 DETs were encoded by chromosome X and were clustered into two expression patterns (Figure 2C).

### GO Enrichment and KEGG Analysis

We analyzed the functional enrichment and significant pathways associated with the DETs clustered in the five expression patterns. The downregulated transcript cluster in most infected tissues (pattern 1) was significantly enriched for GO terms involved in metabolic or tissue development processes, such as proteinaceous extracellular matrix, lipid metabolic process, animal organ development, PPAR signaling pathway, and metabolism of xenobiotics by cytochrome P450 (Supplementary Table 4). In the upregulated transcript clusters in infected liver (pattern 2) and most infected tissues (pattern 5), most of the enriched transcripts were related to GO terms or pathways involved in immune response. These included cytokine receptor binding, regulation of interleukin (IL)-12 production, Jak-STAT signaling pathway, nuclear factor kappa B (NF-κB) signaling pathway, lymphocyte chemotaxis, IL-8 secretion, and cytokine secretion (Supplementary Tables 5, 6). The cluster containing upregulated transcripts at 6 dpi (pattern 4) was not enriched in any GO term. However, most significantly enriched GO terms in the transcript's cluster with high expression pattern at 18 dpi (pattern 3) were related to immune response, such as antigen processing and presentation pathway, lytic vacuole, response to cytokine, lymphocyte-mediated immunity, and chemokine-mediated signaling pathway (Supplementary Table 7). Only five transcripts encoded by X chromosome were involved in lipid metabolic processes (Figure 2C).

**Figure 3:**
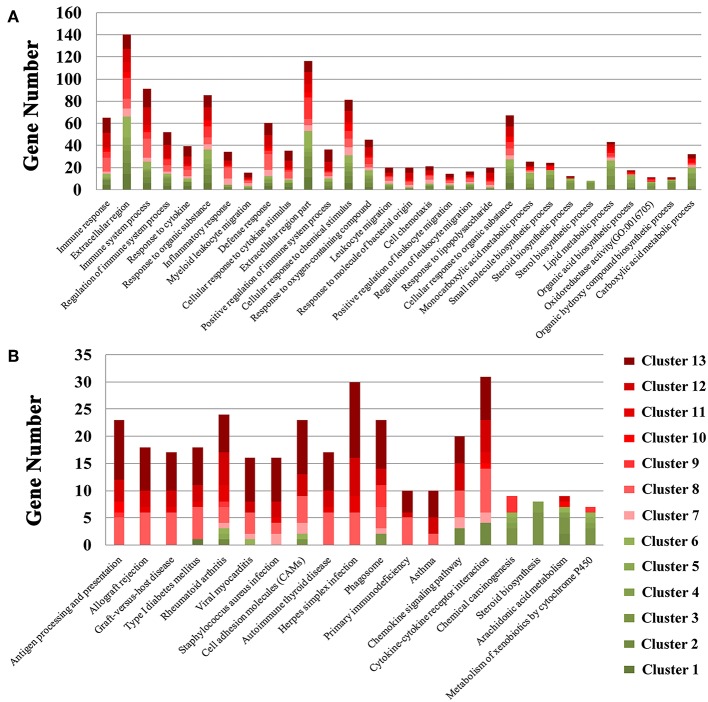
Stacked bar plots showing the results of GO enrichment **(A)** and KEGG analysis **(B)** of genes in the 13 clusters identified in Figure 2A.

**Figure 4:**
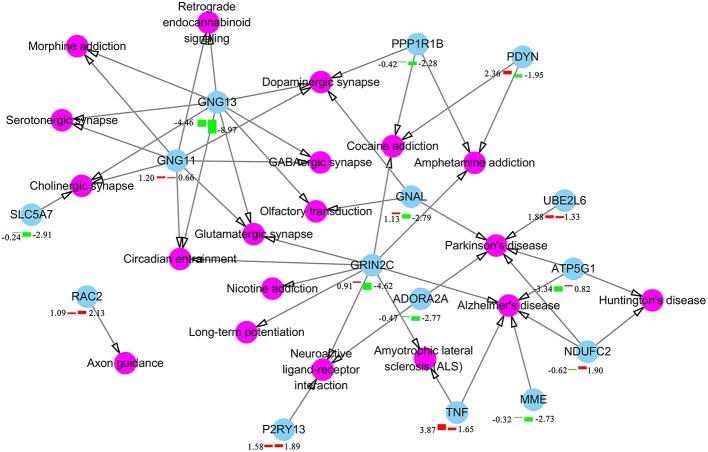
Differentially expressed components of neuron signaling pathways in *T. gondii* infected brain. Pink color represents neuron signaling pathway and cyan color represents the differentially expressed transcripts in brain at 6 or 18 dpi. The bars underneath the nodes represent the change in the expression level (left and right bars represent expressional changes at 6 and 18 dpi, respectively). Upregulated and downregulated transcripts are represented by red and green colors, respectively. The log2 fold-change of transcripts are described together with the bars.

We also performed GO enrichment and KEGG analysis of all DETs. According to FDR corrected *P*-value, the top 30 significantly enriched GO terms and pathways are shown in Figure 3. Most of the transcripts that were significantly enriched in immune-related GO terms or pathways belonged to clusters 7–13, which had upregulated expression patterns (Figure 2A). However, most of the transcripts that were significantly enriched in metabolic related GO terms or pathways belonged to clusters 1–6, which had low expression patterns (Figure 2A). In the infected brains, 19 neural signaling pathways were regulated by 15 DETs, including *Adora2a, P2ry13, Grin2c, Gng11, Gng13, Ppp1r1b, Pdyn, Gnal, Rac2, Atp5g1, Ndufc2, Slc5a7, Ube2l6, Tnf*, and *Mme* (Figure 4).

### Coexpression and Regulatory Network Analysis

**Figure 5:**
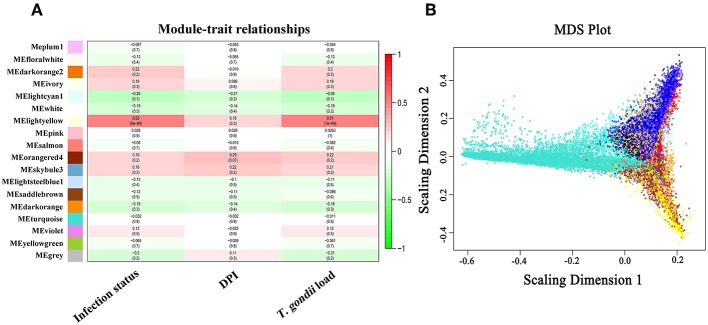
**(A)** Module and traits relationships. The color-coded legend shows the correlation degree, with positive and negative numbers indicating positive and negative relationship, respectively **(B)**. The classical multidimensional scaling plot of all transcripts.

**Figure 6:**
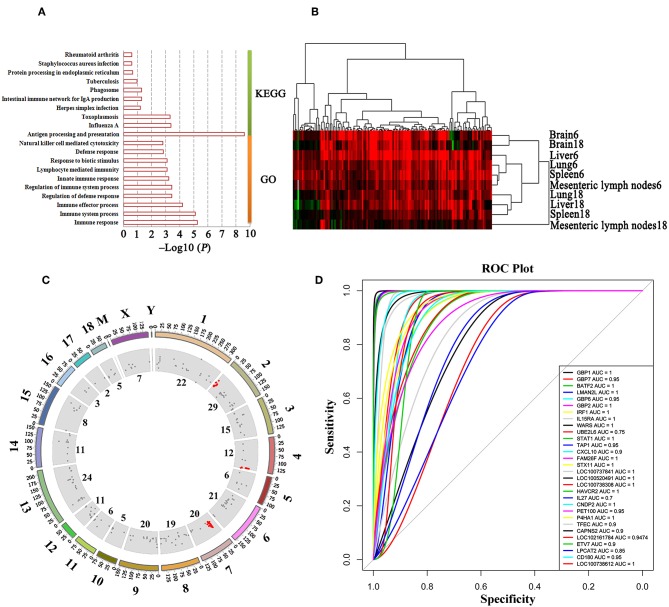
Functional annotation and transcript expressional cluster of MElightyellow module. **(A)** GO and KEGG enrichment of transcripts in MElightyellow module. The top 10 GO terms are listed according to the enrichment *P*-value. **(B)** Expressional clustering of transcripts in MElightyellow module. Upregulated, downregulated, and missing transcripts are denoted by red, green, and gray colors, respectively. **(C)** Distribution of the host genes significantly correlated with *T. gondii* load (HGSCTG) on the pig genome. Outer circle represents the chromosome, scatter in inner circle represents the distribution of HGSCTG, the numbers adjacent to inner circle represent the number of HGSCTG that are encoded by the chromosome, red scatter shows HGSCTG distributed on the hotspot sites. **(D)** ROC tests between the top 30 hub gene expressions and *T. gondii* parasite DNA load.

The soft-threshold power, that exceeded a scale-free topology fit index of 0.75 for each network was 14 (Supplementary Figure 4). Therefore, number 14 was chosen as the soft power threshold for constructing WGCNA. As shown in Figure 5A, 18 modules, including gray module, denoting unassigned transcripts, were found. The module-trait association shows that MElightyellow was significantly correlated with infection status and *T. gondii* load (Figure 5A). The global correlation coefficient between MElightyellow and *T. gondii* load was 0.51 with a *P*-value of 7e-04. Details of the transcripts and module relationships are summarized in Supplementary Table 8. Multidimensional scaling (MDS) plot shows that the transcripts of each module were successfully categorized by coexpression analysis (Figure 5B). Most of the genes in MElightyellow module were upregulated in most infected tissues, and significantly enriched in immune-related biological processes (Figures 6A,B). The genomic locations of genes in the MElightyellow module are shown in Figure 6C.

We identified 152 genes (165 transcripts in total) as HGSCTG that were significantly correlated with *T. gondii* loads. The details of HGSCTG are listed in Supplementary Table 9. Chromosome 2 encodes most of the MElightyellow module genes (29 genes) and these genes were distributed on 3 spots, including head spot (chromosome 2: 0Mb-13Mb), which encodes 10 genes (8 of these were HGSCTG), middle spot (chromosome 2: 53–84 Mb), which encodes 10 genes (8 of these were HGSCTG), and end spot (chromosome 2: 124–162 Mb), which encodes 9 genes (4 of these were HGSCTG). Chromosome 4: 139–144 Mb encodes 6 HGSCTG and chromosome 7: 22–38 Mb encodes 10 HGSCTG. Therefore, chromosome 2: 0.3–13 Mb, chromosome 4: 139–144 Mb, and chromosome 7: 22–38 Mb were identified as HGSCTG genomic hotspots. The details of HGSCTG locations in the hotspots are shown in Table 1. Results of the ROC analysis of the top 30 hub genes of MElightyellow module are shown in Figure 6D. All the areas under ROC curve (AUC) were >0.7 and the majority of them were >0.9, indicating a high correlation between the top 30 hub gene expression and *T. gondii* parasite DNA load. Functions of the hub genes are described in Table 2.

**Table 1:** The genomic location of the host genes significantly correlated with *T. gondii* load (HGSCTG) in the HGSCTG hotspots.

**Gene symbol**	**Chromosome**	**Start site**	**End site**	**Mean RPKM**	**AUC**
IRF7	Chromosome 2	299457	302151	4.7	0.65
LOC102161205	Chromosome 2	2828704	2838699	2.99	0.95
TCIRG1	Chromosome 2	3476372	3488276	1.2	0.65
LOC100736864	Chromosome 2	6230389	6231678	1	1
BATF2	Chromosome 2	6312368	6321030	1.85	1
STIP1	Chromosome 2	6978503	7040367	194	0.7
FAM111A	Chromosome 2	11864850	11873989	1.3	0.8
UBE2L6	Chromosome 2	12987538	13002246	143.7	0.75
LOC102161784	Chromosome 4	139395369	139411664	2.46	0.95
LOC100737841	Chromosome 4	139499543	139509159	2.77	1
LOC100523668	Chromosome 4	139559304	139570934	2.45	1
GBP7	Chromosome 4	139582087	139601514	123.4	0.95
GBP2	Chromosome 4	139612348	139676554	14.9	1
GBP1	Chromosome 4	139651545	139667272	179.1	1
LOC100622791	Chromosome 7	24603720	24604965	2751.5	1
SLA-3	Chromosome 7	24641613	24645323	772.3	1
UBD	Chromosome 7	25330502	25332256	118.5	1
TAP2	Chromosome 7	29412165	29423473	18.1	0.95
TAP1	Chromosome 7	29429379	29438656	5.35	0.95
PSMB9	Chromosome 7	29438820	29443905	46	1
SLA-DMB	Chromosome 7	29485890	29491702	104.3	1
SLA-DMA	Chromosome 7	29500107	29504539	42.7	0.9
ETV7	Chromosome 7	36987742	37008910	15.6	0.9
PIM1	Chromosome 7	37691993	37697470	41.4	0.95

*Analysis is based on the pig reference genome (Sscrofa10.2) and the annotation file (Sscrofa10.2_top_level.gff3)*.

**Table 2:** Description of mouse or human orthologs of the top 30 hub genes of MElightyellow module.

**Gene symbol**	**Mean RPKM**	**q. weighted**	**cor. weighted**	**Gene function**	**References**
STAT1*	400.5	1.93E-10	0.88	Mediation of the production of MHC, NO, and IFN-inducible GTPase family of proteins that function as anti-*T. gondii* factors directly	^m^ (45)
IRF1*	234.47	9.36E-11	0.88	Cis-acting factor of iNOS which is needed for *T. gondii* control	^m^ (46)
BATF2*	1.85	2.3E-11	0.9	Batf2 play regulatory role in CD8α + classical DC development and contributes to anti-*T. gondii in vivo* infection.	^m^ (47)
CXCL10*	692.79	3.62E-10	0.87	CXCL10 is required to maintain T-cell populations and to control parasite replication during chronic ocular Toxoplasmosis	^m^ (48)
GBP2*	14.9	4.11E-11	0.89	Accumulate around the PV of *T. gondii* and contributes to anti-*T. gondii* in mice.	^m^ (49)
GBP1*	179.1	1.31E-11	0.91	Accumulate around the PV of *T. gondii* and contributes to anti-*T. gondii* in mice.	^m^ (50)
GBP6*	21.6	4.11E-11	0.89	GBP6 accumulate around the PV of *T. gondii* and contributes to anti-*T. gondii*.	^m^ (50)
GBP7*	123.4	2.3E-11	0.90	GBP7 accumulate around the PV of *T. gondii* and contributes to anti-*T. gondii*.	^m^ (50)
IL15RA*	10.6	1.14E-10	0.88	IL15 signal pathway contributes to the development of antigen-specific memory CD8+ T cells against *T. gondii*.	^m^ (51)
TAP1*	5.35	2.11E-10	0.88	TAP1 is one subunit of the transporter associated with antigen processing. TAP-1 indirectly regulates CD4+ T cell priming in *Toxoplasma gondii* infection by controlling NK cell IFN-γ production.	^m^ (52)
IL27*	8.16	1.88E-9	0.86	Interleukin 27 regulates Treg cell populations that required to limit *T. gondii* infection-induced pathology.	^m^ (53, 54)
UBE2L6*	143.7	1.44E-10	0.88	Ubiquitination targets *T. gondii* for endo-lysosomal destruction.	^h^ (55)
WARS	2.3	1.34E-10	0.88	WARS is one component of primary defense system against infection.	^m^ (56)
CD180	10.95	6.08E-9	0.84	CD180/MD1 complex is a member of the Toll-like receptor (TLR) family and it plays a crucial role in the response of immune cells to LPS.	^h^ (57)
LMAN2L	29.77	2.63E-11	0.9	Export of glycoproteins	^h^ (58)
HAVCR2	2.5	1.88E-9	0.86	Positive regulation of tumor necrosis factor secretion	^h^ (59)
LPCAT2	4	5.63E-9	0.85	LPCAT2 is a critically important enzyme for the biogenesis of proinflammatory lipid mediator (Platelet-activating factor) and the membrane homeostasis of inflammatory cells. It can also catalyze the formation of phosphatidylcholine.	^h^ (60, 61)
CNDP2	4	1.98E-9	0.86	CNDP2 is a cytosolic non-specific dipeptidase	^h^ (62)
STX11	55.4	1.14E-9	0.86	In human, STX11 is part of the cytolytic machinery of T and NK cells and involved in the fusion of lytic granules with the plasmamembrane.	^h^ (63)
FAM26F	41.3	4.61E-10	0.87	FAM26F contains an immunoglobin (Ig) like fold and it is expressed on various immune cells, playing a role in infection and immunity.	^h^ (64)
PET100	17	2.05E-9	0.85	PET100 is a molecular chaperone required for the assembly of cytochrome c oxidase.	^h^ (65)
TFEC	4.6	4.49E-9	0.85	TFEC is specifically induced in bone marrow-derived macrophages upon stimulation with the Th2 cytokines.	^m^ (66)
P4HA1	26.7	4.03E-9	0.85	P4HA1 residing within the lumen of the endoplasmic reticulum (ER) and in charge of catalyzing formation of 4-hydoxyproline in collagens. It is essential for mice survival.	^m^ (67)
LOC100738612	21.3	7.25E-09	0.85	Also known as ATF3. Negative regulation of iNOS expression and NO production in activated macrophages to avoid pathogenesis of tissue damage.	^m^ (68)
ETV7	15.66	5.59E-09	0.85	ETV7 is a transcription factor and involved in proliferation and survival of normal mouse B cells.	^m^ (69)
CAPNS2	1.2	4.49E-09	0.85	CAPNS2 is one of cysteine proteases.	^m^ (70)
LOC100737841	2.77	1.18E-09	0.86	LOC100737841 is guanylate-binding protein 2-like protein	^p^ NCBI annotation
LOC100738308	4	1.35E-09	0.86	LOC100738308 is signal transducer and activator of transcription 1-like protein	^p^ Pig genomic annotation file
LOC100520491	98.3	1.31E-09	0.86	According to PSI-BLAST, it homologous to rat Klrb1b which involved in anti-malaria.	^p^ PSI-BLAST and EggNOG 4.5 annotation
LOC102161784	2.46	4.99E-09	0.85	Guanylate-binding protein.	^p^ PSI-BLAST and EggNOG 4.5 annotation

* *Indicates function has been confirmed in mice or human*.

q. Weighted: Weighted p-value corrected with FDR;

*cor. Weighted: correlation of T. gondii load with gene expression weighted by a network term*.

*m: denotes annotation from mice*.

*h: denotes annotation from human*.

*p: denotes annotation from pig*.

### Differentially Expressed Transcription Factors (TFs) and Their Regulatory Networks

**Figure 7:**
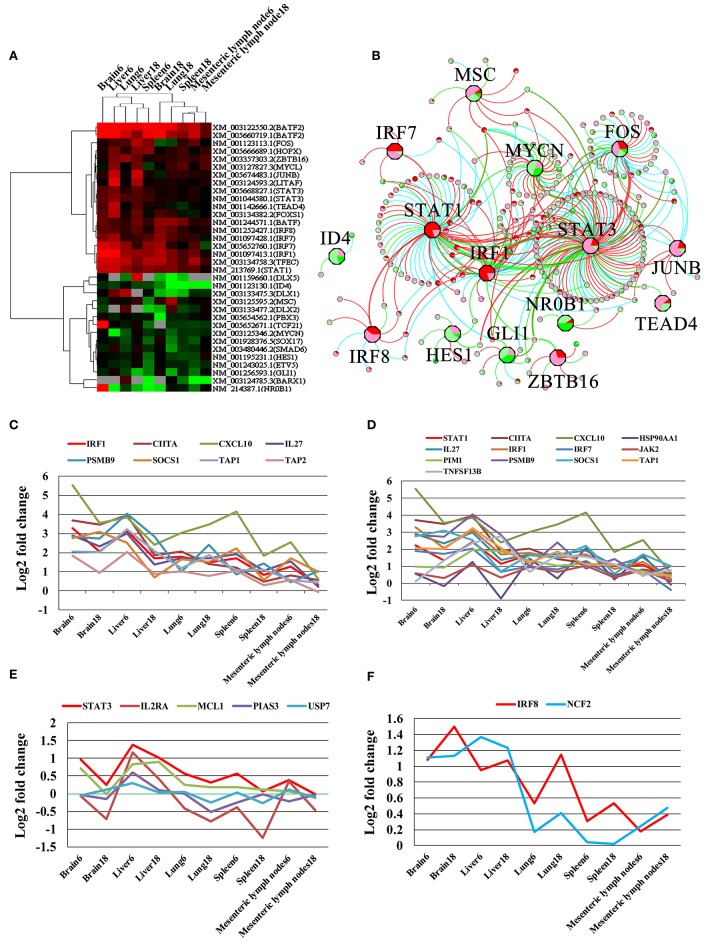
Differentially expressed transcription factors (TFs) and their gene targets. **(A)** Cluster of all differentially expressed TFs. Upregulated, downregulated, and missing transcripts are denoted by red, green, and gray colors, respectively. **(B)** Differentially expressed TFs and their target genes. The octagon nodes represent TF and the circular nodes represent target gene of TF with the pie charts inside the nodes denoting the proportion of expressional change in infected samples. Pie chart colors represent the levels of gene expressional changes: red (log2 fold-change ≥ 1), green (log2 fold-change ≤ −1), pink (log2 fold-change between 0 and 1), light green (log2 fold-change between 0 and −1). **(C–F)** Co-expressed target genes of Irf1, Stat1, Stat3, and Irf8, respectively.

We detected 31 TFs in the infected brain, liver, lung, spleen, and MLN. These 31 TFs were grouped into 2 clusters (Figure 7A). The upregulated TF cluster included *Fos, Hopx, Zbtb16, Mycl, Junb, Litaf*, *Stat3, Tead4, Foxs1, Batf*, *IRF8, BATF2, IRF7, IRF1, Tfec*, and *Stat1*. The downregulated TF cluster included *Msc, Id4, Dlx5, Dlx1, Dlx2, Pbx3, Tcf21, Mycn, Sox17, Smad6, Hes1, Etv5, Gli1, Barx1*, and *Nr0b1*. We found the target genes to 18 TFs in the TRRUST database, including *Stat3, Stat1, Fos, Irf1, Mycn, Gli1, Junb, Msc, Irf8, Hes1, Zbtb16, Irf7, Nr0b1, Dlx5, Id4, Hopx, Tcf21*, and *Tead4*. According to TRRUST database, 240 DETs were regulated by 15 differentially expressed TFs (Figure 7B). As shown in Figures 7C–F, Irf1 was co-expressed with and may function as a regulator for seven target genes (*Cxcl10, Ciita, Il27, Psmb9, Socs1, Tap1*, and *Tap2*); Stat1 was co-expressed with 12 target genes (Cxcl10, Ciita, Il27, Psmb9, Socs1, Tap1, Hsp90aa1, Jak2, Pim1, Tnfsf13b, Irf7, and Irf1); Irf8 was co-expressed with *Ncf2*; and Stat3 was co-expressed with *Il2ra, Mcl1, Pias3*, and *Usp7*.

### Cytokine Expression

**Figure 8:**
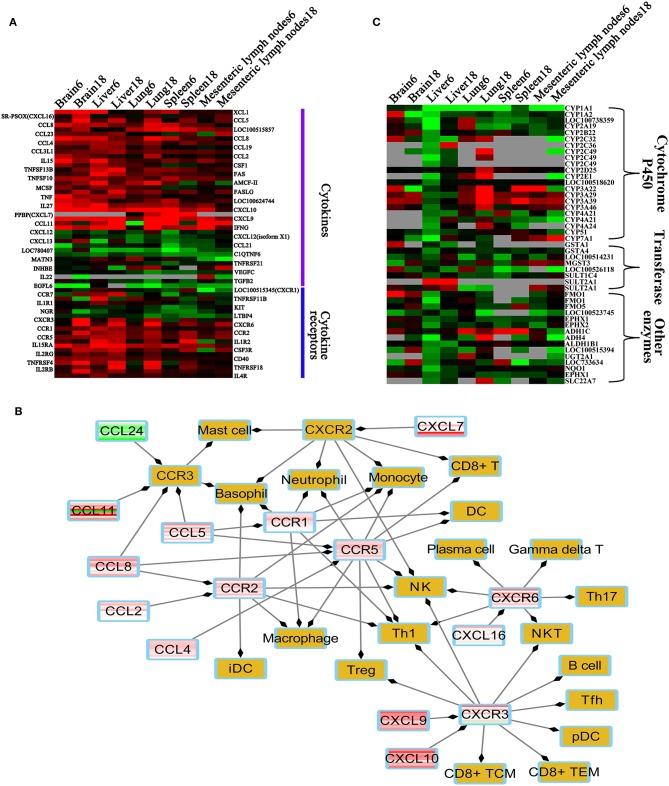
Heatmaps of differentially expressed metabolic enzymes, cytokine and cytokine receptors. **(A)** Heatmap of the differentially expressed cytokines and cytokine receptors. **(B)** Immune cells regulated by differentially expressed cytokines or cytokine receptors. **(C)** Heatmap of differentially expressed enzymes involved in metabolism. Red, green, and gray colors represent upregulated genes, downregulated genes, missing genes, respectively. Brown color denotes non-differentially expressed chemokine receptors and immune cells. Abbreviations: Th, T helper cell; Treg, regulatory T cell; iDC, immature dendritic cell; DC, dendritic cell; pDC, plasmacytoid dendritic cell; NK, natural killer; NKT, natural killer T cell; TCM, central memory T cell; TEM, effector-memory T cell; Tfh, T follicular helper cell.

We detected 38 cytokines and 21 cytokine receptor-related transcripts that were differentially expressed, including 18 differentially expressed chemokines and seven differentially expressed chemokine receptors. Most of these were upregulated in the infected tissues (Figure 8A), including four HGSCTG (*Cxcl10, Il27, Il15*, and *Il15ra*). In infected tissues, upregulation of chemokines and chemokine receptors increases the chemotaxis of 20 immune cells, such as DC, NK, macrophage, and T cells (Figure 8B).

### Comparative Toxicogenomic Analysis

We found that 45 DETs were involved in xenobiotics or drug metabolism (Figure 8C). Most of these were downregulated, especially in the liver at 6 dpi. We also found that the DETs encode enzymes that metabolize 330 substances or drugs, such as ethanol, acetaminophen, ketoconazole, phenobarbital, and benzyl alcohol. The relationship among 330 xenobiotic substances and DETs related to metabolism are shown (Supplementary Table 10).

## Discussion

We compared the transcriptomes of *T. gondii*-infected and uninfected pigs using RNA-seq approach. Hundreds of transcripts were differentially expressed in the porcine brain, liver, lung, spleen, and MLNs at 6 and 18 dpi (Figure 1). These DETs were distributed on all the pig chromosomes, but not the mitochondrial genome and chromosome Y (Figure 2B).

We tested whether transcriptomic changes overlap across porcine tissues. As shown in Figure 1C, there was no common DET in the 10 tissue groups. Next, we characterized the transcriptomic changes in infected tissues using gene clustering analysis, which showed that all DETs are clustered into five different expressional patterns (Figure 2A). Functional enrichment analysis of the downregulated transcripts in most infected tissues revealed that downregulation of metabolism-related and tissue development-related transcripts is prominent in infected tissues (Supplementary Table 4 and Figure 3). This finding may have clinical relevance. During pregnancy, mother-to-fetus transmission of *T. gondii* can occur, resulting in abortion, stillbirth, or congenital malformation (71). It remains to be determined if the downregulation of these transcripts observed in pigs can also occur in the fetus if they become congenitally infected. Mindful of the fact that successful pregnancy requires delicate immune balance to protect the fetus, the deleterious effects of *T. gondii* induced-inflammatory response mediated by increased expression of cytokines and cytokine receptor-related transcripts (Figure 8A and Supplementary Table 6) may cause undesirable health consequences in the fetus.

Forty-three genes involved in lipid metabolic processes, including 26 genes belonging to clusters 1–6 (Figure 3A) showed lower expression in infected tissues (Figure 2A). Five of these were encoded by chromosome X (Figure 2C). These results suggest that, during *T. gondii* infection, chromosome X contributes ~ one-eighth of the downregulated genes involved in lipid metabolism. The downregulation of metabolic terms or pathways is consistent with our previous proteomic and transcriptomic investigations in mice (72–74), suggesting that downregulation of metabolic processes may also occur in other mammalian hosts.

*T. gondii* influences mouse behavior via altering glutamate transporter Slc1a2 (also known as GLT-1) (75) and GABA signaling pathway (76). In our study, *Slc1a2* was not differentially expressed in the brain. However, three genes (*Gng11, Gng13*, and *Grin2c*) involved in the signaling mechanism of glutamatergic and GABAergic synapse were differentially expressed. This indicates that *T. gondii* may alter pig behavior by interfering with glutamatergic and GABAergic synapse pathways via altering the expression of *Gng11, Gng13*, and *Grin2c*. We also found 19 neural synapse signaling pathways altered in infected brains (Figure 4), such as olfactory transduction pathway, which may alter the sense of smell of infected pigs. Chronically infected rodents exhibit behavioral changes, such as loss of aversion and even attraction to cat odors (77). In humans, infection with *T. gondii* has been associated with behavioral abnormalities (78) and increased risk of developing psychiatric disorders (79).

We further investigated which host genes are significantly correlated with *T. gondii* load (HGSCTG) using WGCNA analysis, which identified 18 coexpression modules (Figure 5 and Supplementary Table 8). By relating modules to sample traits (days after infection, infection status, and *T. gondii* load in tissues), MElightyellow module was significantly correlated with parasite load in infected tissues (Figure 5A). Most of the transcripts in MElightyellow module were upregulated in infected tissues (Figure 6B). As shown in Figure 6A, genes in MElightyellow module were significantly enriched in immune response and infection-related terms or pathways. By combining WGCNA coexpression and ROC curve analyses, we identified 152 HGSCTG (Supplementary Table 9), including three HGSCTG cytokines (*Cxcl10, Il27, Il15*) and one cytokine receptor (*Il15ra*). These four genes seem to be important for mice survival during *T. gondii* infection (Table 2).

Three HGSCTG genomic hotspots (Figure 6C) encoding 24 HGSCTG (Table 1) were also identified. Most of these 24 genes were involved in immune processes, and some have anti-*T. gondii* activity, such as *Gbp1, Gbp2, Gbp7, Batf2*, and *Tap1*. As shown in Figure 6D, the AUC of the top 30 hub genes in the MElightyellow module was >0.7 and for most of these genes, the AUC was >0.9, indicating a strong correlation between the top 30 hub gene expression and *T. gondii* DNA load. This result shows a synergy between the results obtained by ROC and WGCNA analysis, suggesting that the identified hub genes (*CD180, STX11, FAM26F, TFEC, ETV7, LOC100738612*, and *LOC100520491*) are HGSCTG.

We detected 31 differentially expressed TFs, grouped into upregulated and downregulated clusters (Figure 7A). In these differentially expressed TFs, Batf2, Irf7, Irf1, Tfec, and Stat1 were HGSCTG. We also found hundreds of gene targets to 15 differentially expressed TFs in the TRRUST database (Figure 7B). The coexpression analysis revealed that 12, 7, 1, and 4 targeted genes shared similar expression pattern with Stat1, Irf1, Irf8, and Stat3, respectively (Figure 7). The Irf1 and Stat1 contribute to *T. gondii* control via regulating the expression of factors essential for host resistance to infection, such as TAP complex (80), Cxcl10, Ciita (81), Il27, and Jak2 (82). We also identified 38 cytokines and 21 cytokine receptor-related transcripts that were differentially expressed. Most of these were upregulated in infected tissues (Figure 8A). In agreement with others (83), upregulation of these genes can increase chemotaxis of 20 immune cells, including DCs, NK cells, macrophages, and T cells in most infected tissues (Figure 8B). These immune cells, which play important roles in *T. gondii* control (6), can be regulated by CXCl9, CXCl10, and CXCR3 signaling pathways (Figure 8B), contributing to the pig immune response to *T. gondii* infection.

As shown in Figure 8C, 45 DETs were involved in xenobiotics or drug metabolism, most of these were downregulated in infected tissues, especially in the liver at 6 dpi. According to the DrugBank database, 330 xenobiotics or drugs were found to be metabolized by enzymes coded by these DETs (Supplementary Table 10). Acetaminophen is used to control fever, however, it can induce adverse events, such as acute liver failure (84). In our study, four downregulated genes (*Cyp1a2, Cyp2e1, Cyp1a1*, and *Sult2a1*) were involved in the pharmacokinetic of acetaminophen. The downregulation of metabolic transcripts are alleviated in infected livers at 18 dpi (Figure 3), indicating that downregulation of metabolic processes in infected liver varies by stage of infection and that acute *T. gondii* infection causes more inhibition of the hepatic metabolic processes. This result is consistent with previous work showing downregulation of genes involved in liver metabolism following *T. gondii* infection (72, 73).

Downregulation of xenobiotics or drug metabolism in liver is related to inflammatory response (85). Although transcripts in the cluster showing higher expression in most infected tissues were significantly enriched in immune-related terms and pathways (Supplementary Table 5), transcripts in the high expression pattern in the liver cluster (Figure 2A) were also significantly enriched in terms related to inflammatory processes (Supplementary Table 6). This suggests that liver exhibited more inflammatory response than other tissues. Likewise, transcripts in the cluster with high expression pattern at 18 dpi were significantly enriched in GO terms related to immune responses (Supplementary Table 7). The ability of *T. gondii* to cause severe hepatic pathologies has been demonstrated (86, 87), and it is possible that the prominent inflammatory response observed in our study contributes to the pathologies observed in infected livers.

## Conclusion

RNA-seq was used to determine the global changes in the porcine transcriptome subsequent to *T. gondii* infection at 6 and 18 days post infection. Hundreds of DETs exhibited differential expression profiles in infected tissues and were clustered into five expression patterns. Infection induced downregulation of various metabolic processes in most infected tissues, especially in the liver during acute infection. The WGCNA analysis showed that, *T. gondii* infection causes differential expression of transcription factors, such as Irf1, Irf8, Stat1, and Stat3. We also identified 45 DETs encoding detoxifying enzymes involved in the metabolism of 300 xenobiotics or drugs. These data improve our understanding of the molecular changes that occur during *T. gondii* infection in pigs. Although results obtained in pigs may not be readily transferrable to humans, given the physiological and immunological similarities between pigs and humans, our findings may facilitate the understanding of how humans might respond to *T. gondii* infection.

## Ethics Statement

The study design was reviewed and approved by the Animal Ethics Committee of Lanzhou Veterinary Research Institute (LVRI), Chinese Academy of Agricultural Sciences (CAAS). The procedures involving animals were carried out in accordance with the Animal Ethics Procedures and Guidelines of the People's Republic of China. Animals were monitored every day for the development of clinical signs of toxoplasmosis. All efforts were made to minimize suffering and to reduce the number of pigs used in the experiment.

## Author Contributions

X-QZ, HE, and J-JH conceived and designed the study and critically revised the manuscript. J-JH performed the experiment, analyzed the transcriptomic data, and drafted the manuscript. JM and HE helped in study design, implementation, data analysis, and manuscript revision. J-LW, F-KZ, J-XL, B-TZ, and Z-XW helped in the study implementation. All authors have read and approved the final manuscript.

### Conflict of Interest Statement

The authors declare that the research was conducted in the absence of any commercial or financial relationships that could be construed as a potential conflict of interest.

## Supplementary Material

Supplementary Figure 1 Sequencing qualities of the uninfected and infected tissues at 6 dpi. The darker the color the better the global sequencing quality.

Supplementary Figure 2 Sequencing qualities of the uninfected and infected tissues at 18 dpi. The darker the color the better the global sequencing quality.

Supplementary Figure 3 Distribution of sequencing qualities. Vertical axis represents the percentage of clean reads with sequencing quality > Q20. Horizontal axis represents the samples sequenced in the present study.

Supplementary Figure 4 Scale-free topology fit index of coexpression analysis. Scale independence map shows the relationship between soft power and scale free topology model fit of WGCNA analysis. Mean connectivity map shows the relationship between soft power and mean connectivity which summarizes the connection strengths with other genes.

Supplementary Table 1 Primers used for quantitative reverse transcriptase qRT-PCR assay to validate the RNA-seq data.

Supplementary Table 2 Normalized *Toxoplasma gondii* DNA load in infected pig tissues.

Supplementary Table 3 Differential expression profiles of transcripts across various tissues of the pigs.

Supplementary Table 4 Significantly enriched GO terms and pathways of DETs in the lower expression cluster in most infected tissues.

Supplementary Table 5 Significantly enriched GO terms and pathways of DETs in the higher expression cluster in most infected tissues.

Supplementary Table 6 Significantly enriched GO terms and pathways of DETs in the higher expression cluster in infected liver.

Supplementary Table 7 Significantly enriched GO terms and pathways of DETs in the higher expression cluster at 18 dpi.

Supplementary Table 8 Modules of coexpressed genes.

Supplementary Table 9 Details of the host genes significantly correlated with *T. gondii* load (HGSCTG).

Supplementary Table 10 Relationship between 330 xenobiotics and differentially expressed metabolism related genes.
