# Supplementary figures and images for: Global Transcriptome Profiling of Multiple Porcine Organs Reveals Toxoplasma gondii-Induced Transcriptional Landscapes
Source: Front Immunol. 2019 Jul 3;10:1531. doi: 10.3389/fimmu.2019.01531 (PMC6618905; doi:10.3389/fimmu.2019.01531)

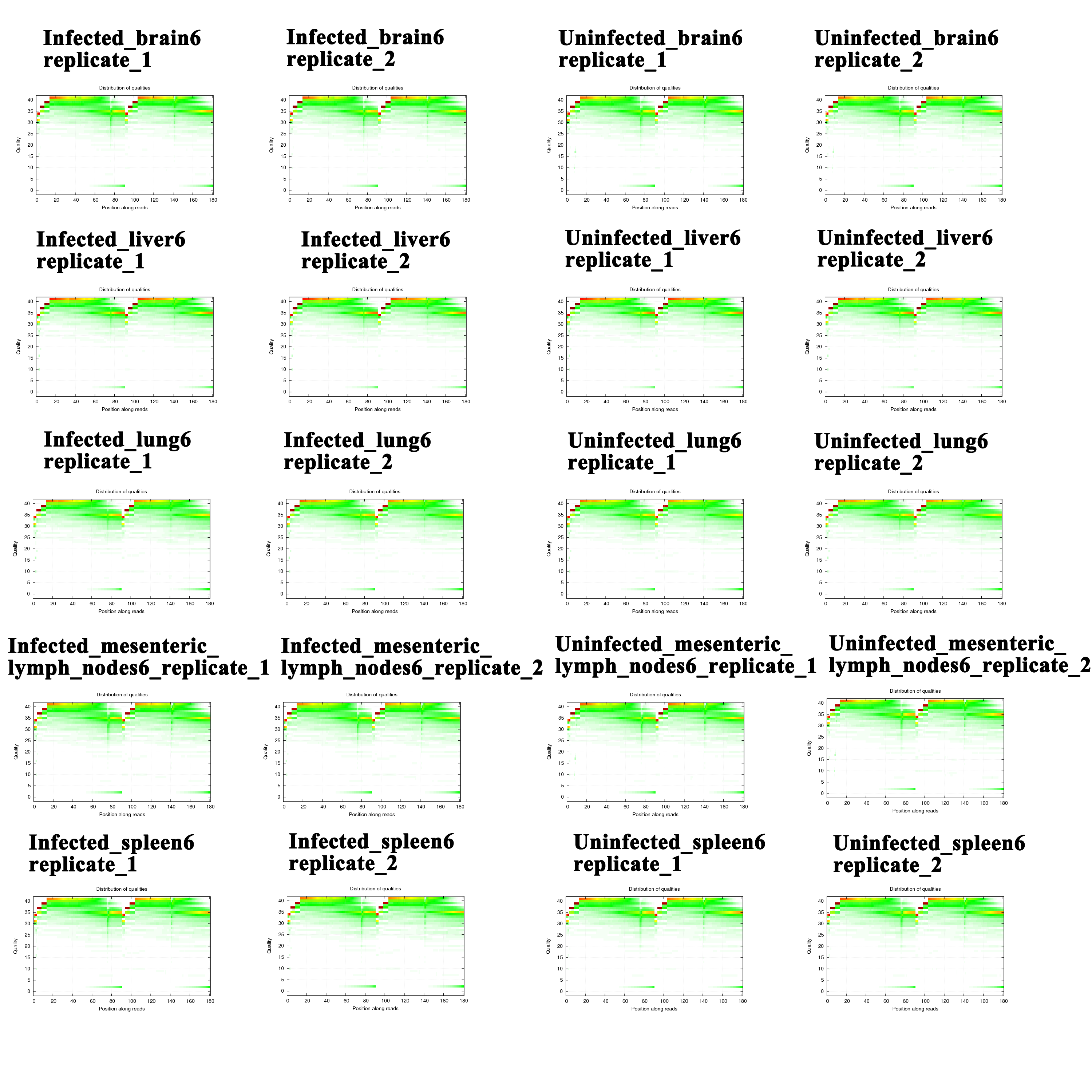

Supplement: Supplementary Figure 1 — Sequencing qualities of the uninfected and infected tissues at 6 dpi. The darker the color the better the global sequencing quality. [file Image_1.TIF]

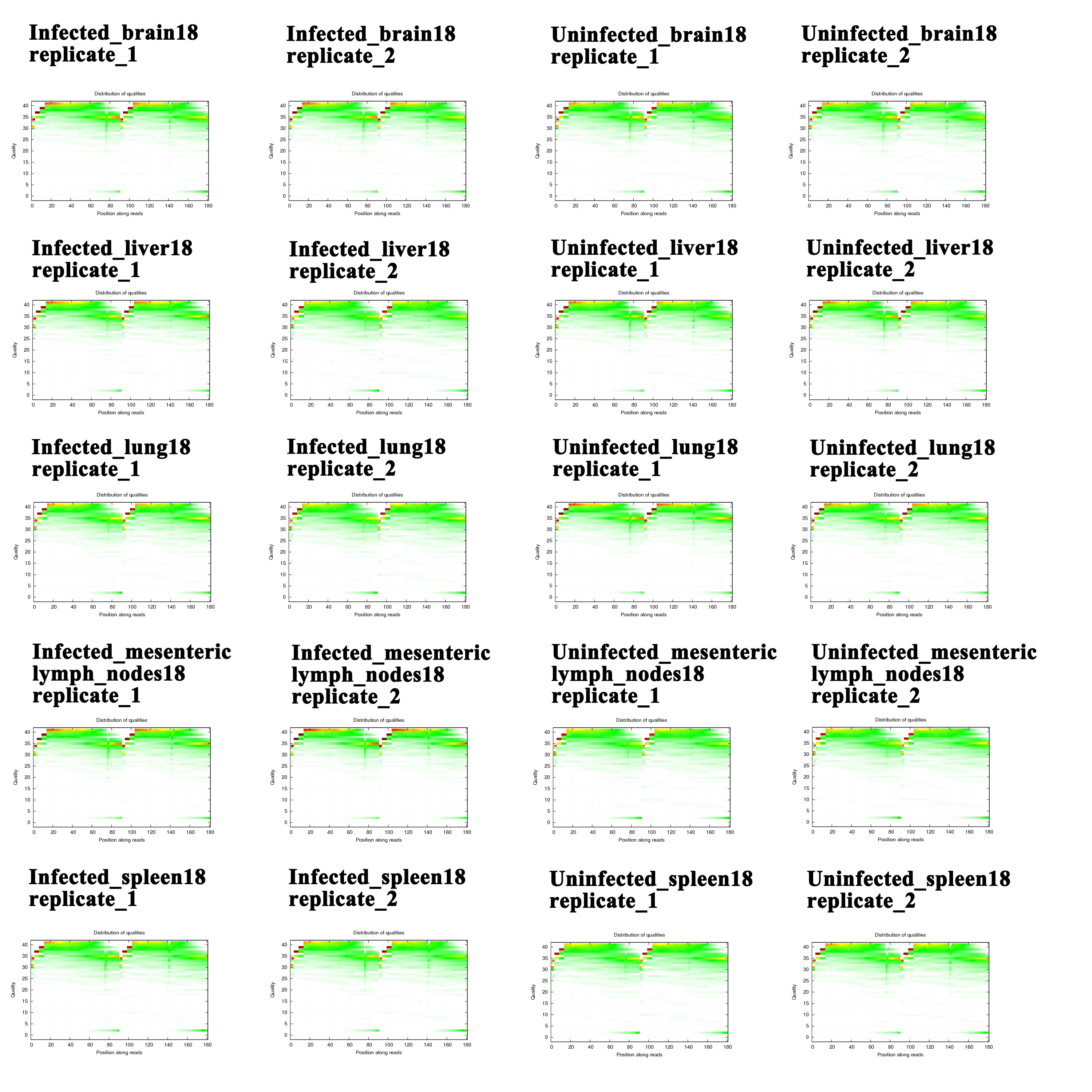

Supplement: Supplementary Figure 2 — Sequencing qualities of the uninfected and infected tissues at 18 dpi. The darker the color the better the global sequencing quality. [file Image_2.TIF]

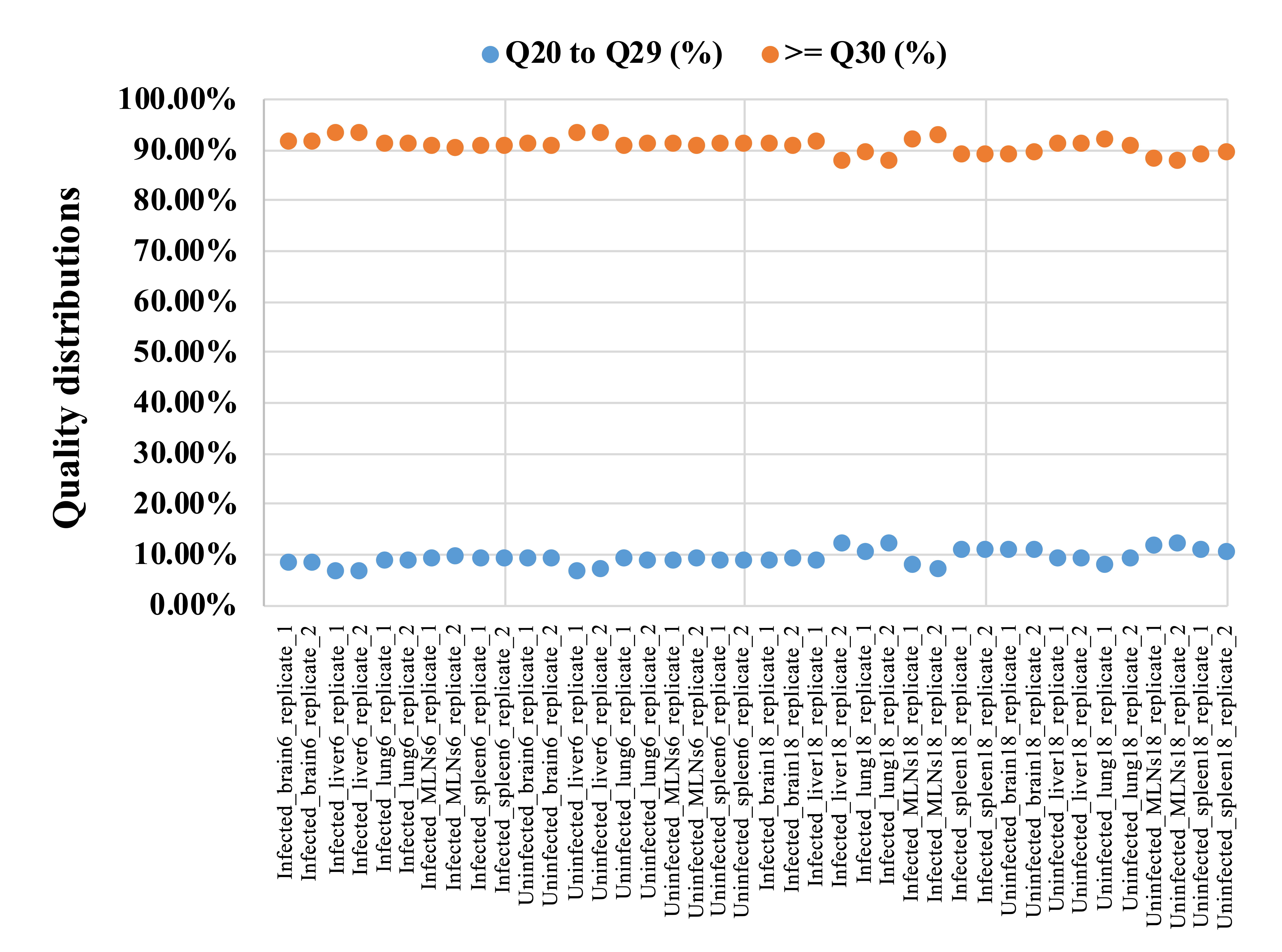

Supplement: Supplementary Figure 3 — Distribution of sequencing qualities. Vertical axis represents the percentage of clean reads with sequencing quality > Q20. Horizontal axis represents the samples sequenced in the present study. [file Image_3.TIF]

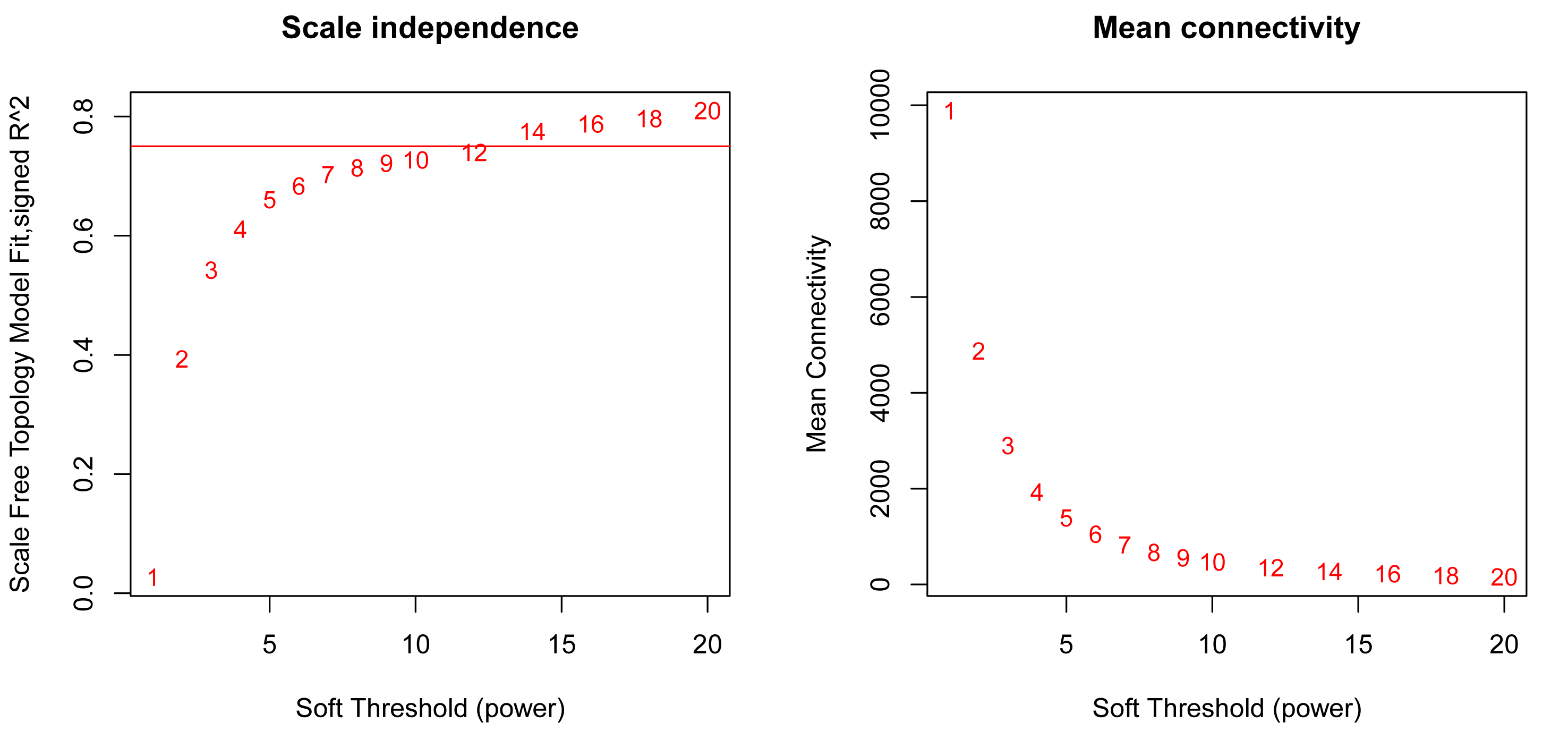

Supplement: Supplementary Figure 4 — Scale-free topology fit index of coexpression analysis. Scale independence map shows the relationship between soft power and scale free topology model fit of WGCNA analysis. Mean connectivity map shows the relationship between soft power and mean connectivity which summarizes the connection strengths with other genes. [file Image_4.TIF]
