# Supplementary material for: Global Transcriptome Profiling of Multiple Porcine Organs Reveals Toxoplasma gondii-Induced Transcriptional Landscapes
Source: Front Immunol. 2019 Jul 3;10:1531. doi: 10.3389/fimmu.2019.01531 (PMC6618905; doi:10.3389/fimmu.2019.01531)
Supplement: Supplementary Table 1 — Primers used for quantitative reverse transcriptase qRT-PCR assay to validate the RNA-seq data. [file Table_1.DOCX]

**Table S1.** The qRT-PCR primers used for RNA-seq validation

| **Gene** | **Primer F** | **Primer R** | **Access Number** |
| --- | --- | --- | --- |
| SLPI | ATGTGCGGGAAAGTCTGC | CTGGGAGTGGAAATGGAA | NM_213870.1 |
| TNFSF18 | GCCATTCAAGTCCTCAC | GGTCCAAACTTCGCTAC | XM_005667782.1 |
| CXCL12 | CCTTGCCGATTCTTTGAG | AGTGGGACTGGGTTTGTTT | NM_001009580.1 |
| IL1R2 | GAAATCCCTGGACACGA | GAATGACGAAGGCAGAA | NM_001243363.1 |
| MYCL | AGCCAGTCACCATCACG | TCTGCCTCCTCTTCCTTT | XM_003127827.3 |
| KLHDC8A | CCTGATGGGAGTGAGACG | ATGCCACAGTGTAAATGAGAA | XM_003357392.2 |
| ACOT12 | CCCAGAATCAAACCCAT | CCTCCACATTGCCATTAC | XM_003480872.2 |
| UBE2L6 | CTCCTGCCTGAGAAACCG | CACCTGGCCTTCGCTGT | NM_001246215.1 |
| LOC100737768 | GACCTACACGCCCACAA | GCTCCTGCTTAACGGTATTT | XM_003481084.2 |
| TGFB2 | AAAACAGTGGGAAGACC | CTGCTATGCTGGGTGTC | XM_005653762.1 |
| CD79B | CATCATCGTGCCCATCTT | ACCCACCGACCACTTCA | NM_001243912.1 |
| IRG1 | GCTGCTTGGTAAGGTGG | GGCATTGGCTCTGAACT | XM_003131032.1 |
| GPR1 | ATAACAACTTCCACGAGC | GAGTCCAGCAAATCAAA | NM_001190244.1 |
| TM7SF4 | AGTGACGGCGACAGAGGT | CTGCTTATTCAGCGGGAG | NM_001185150.1 |
| ID4 | GCCGCACGTTATTGACTACATC | TGTCGCCCTGCTTGTTCACC | NM_001123130.1 |
| CCL5 | AAGAAATGGGTGCGGGAGT | CTGGGACAAGAGCAAGAAGC | NM_001129946.1 |
| TAP1 | CAGATCCAGTTCACCGAAGC | GGGTAGGCAAAGGAGACAT | NM_001044581.1 |
| ANGPT1 | ACTTTGGTAGCCAGATGA | TATTGTTAGTGGGTTTGC | NM_213959.1 |
| CCL21 | CAAGGCAGTGATGGAGGAG | AACAGGATAGCTGGGATGG | NM_001005151.1 |
| IL10 | GCATCCACTTCCCAACCA | GCAACAAGTCGCCCATCT | NM_214041.1 |
| IFNG | AAATGGTAGCTCTGGGAAAC | TATTGCAGGCAGGATGAC | NM_213948.1 |
| CYP1A2 | TATCTGCCCAGCCCTAC | TGTCCTGGATACAGTTCTTG | XM_005666124.1 |
| ADAMTS7 | GGTTTCCGAGGATAATGACG | TGGGACCTGAAGAGTGGG | XM_005656262.1 |
| IL27 | TTTTGCTGCTTTCCTTGCT | GCTGACCTTGAACTCCCTCT | NM_001007520.1 |
| ACTG2 | TATGAGGGATACGCACTG | GATGTCTCGCACGATTT | XM_003125028.4 |
| ZBP1 | CTGTCAGAAGGGACTGAACAAC | CGTCGGAGGGAGGTAGAA | NM_001123216.1 |
| LAMB2 | GGTCTGTGAGTCCCGCTAT | ACCCGTAGGTTGGTAATCTTT | XM_005669535.1 |
| ASNS | ACATTCGGAAGAACACGG | CACGGAGAACATCAAACAAA | NM_001167640.1 |
| CYP2B22 | GAGATCGACCGGGTGATT | CTCGGAAGTGAGTGTCTTTAGT | NM_214413.1 |
| COL1A1 | CCGAAGGCGAGGTGTCA | TCCAGGAAGTCCAGGTTGT | XM_005668927.1 |
| CASP4 | GGAGATGGAATCGGTGCT | GAGGCAGTTGCGGTTGT | XM_005667286.1 |
| IL1B | ACCTGGACCTTGGTTCTC | GGATTCTTCATCGGCTTC | NM_214055.1 |
| GAPDH | GGTGAAGGTCGGAGTG | TGATGTTGGCGGGAT | NM_001206359.1 |
