# Supplementary material for: Global Transcriptome Profiling of Multiple Porcine Organs Reveals Toxoplasma gondii-Induced Transcriptional Landscapes
Source: Front Immunol. 2019 Jul 3;10:1531. doi: 10.3389/fimmu.2019.01531 (PMC6618905; doi:10.3389/fimmu.2019.01531)
Supplement: Supplementary Table 2 — Normalized Toxoplasma gondii DNA load in infected pig tissues. [file Table_2.DOCX]

| Samples | Days postinfection | Normalized *T. gondii* load (Log2( *T. gondii* B1gene abundance/pig 18S abundance)) |  |
| --- | --- | --- | --- |
| Infected_brain6_replicate_1 | 6 | -13.48 |  |
| Infected_brain6_replicate_2 | 6 | -13.265 |  |
| Infected_liver6_replicate_1 | 6 | -15.6233 |  |
| Infected_liver6_replicate_2 | 6 | -14.9933 |  |
| Infected_lung6_replicate_1 | 6 | -12.9167 |  |
| Infected_lung6_replicate_2 | 6 | -12.9567 |  |
| Infected_MLNs6_replicate_1 | 6 | -13.8267 |  |
| Infected_MLNs6_replicate_2 | 6 | -14.0067 |  |
| Infected_spleen6_replicate_1 | 6 | -17.5567 |  |
| Infected_spleen6_replicate_2 | 6 | -16.69 |  |
| Infected_brain18_replicate_1 | 18 | -12.36 |  |
| Infected_brain18_replicate_2 | 18 | -12.9933 |  |
| Infected_liver18_replicate_1 | 18 | -14.4 |  |
| Infected_liver18_replicate_2 | 18 | -14.2567 |  |
| Infected_lung18_replicate_1 | 18 | -11.4833 |  |
| Infected_lung18_replicate_2 | 18 | -11.3367 |  |
| Infected_MLNs18_replicate_1 | 18 | -18.5567 |  |
| Infected_MLNs18_replicate_2 | 18 | -18.38 |  |
| Infected_spleen18_replicate_1 | 18 | -15.3033 |  |
| Infected_spleen18_replicate_2 | 18 | -15.6833 |  |
| Abbreviation: MLNs, mesenteric lymph nodes. | | | |

**Table S2**. *Toxoplasma gondii* DNA load in pig tissues.
